# Factors influencing utilization of healthcare services for internal migrant older adults in Xuzhou, China: based on Anderson’s model

**DOI:** 10.3389/fpubh.2024.1378790

**Published:** 2024-11-21

**Authors:** Guiyuan Li, Zijian Qi, Wenxin Yu, Qingzhi Wang, Hao Hou, Chunxia Miao, Wenjun Yan, Xiuyin Gao

**Affiliations:** ^1^Department of Social Community and Health Education, School of Public Health, Xuzhou Medical University, Xuzhou, Jiangsu, China; ^2^Huadong Research Institute for Medicine and Biotechniques, Nanjing, Jiangsu, China; ^3^Department of psychiatry, Renmin Hospital of Wuhan University, Wuhan, Hubei, China; ^4^Department of health management, School of Management, Xuzhou Medical University, Xuzhou, Jiangsu, China

**Keywords:** migrant older adults, outpatient services, inpatient services, Anderson’s model, influencing factors

## Abstract

**Background:**

As population migration increases and the aging process accelerates, the number of migrant older adults is growing significantly. This trend presents a substantial challenge to urban health services in regions experiencing an influx of older adults individuals. Consequently, examining the healthcare service utilization of the migrant older adults population is crucial to promote healthy aging.

**Methods:**

A multi-stage random sampling method was employed to select a study population of 568 migrant older adults individuals, aged 60 years and above, residing in urban Xuzhou City. Multivariate logistic regression analysis, based on Anderson’s model, was conducted to explore the factors influencing outpatient and inpatient health service utilization among this population.

**Results:**

Among the 568 migrants, 73 (12.9%) had received outpatient services within the past 2 weeks, while 109 (19.2%) had received inpatient services within the past year. Migrant older adults with education level, time to health centers, and self-assessed health status negatively influenced the outpatient services utilization among migrant older adults. Possession of pension insurance, convenience to visit, sick for the past 2 weeks, and health services need positively influenced the outpatient services utilization among migrant older adults. Furthermore, age, possession of pension insurance and health insurance, convenience to visit, number of chronic diseases, sick for the past 2 weeks, and healthcare service need positively influenced inpatient service utilization among migrant older adults. Education level, self-assessed health status, and time to health centers negatively influenced the inpatient services utilization among migrant older adults.

**Conclusion:**

The overall utilization of healthcare services by migrant older adults in Xuzhou remains inadequate. Addressing this issue requires enhanced medical policy support and assistance, stronger health education initiatives, and improved social integration for the older adults. Additionally, efforts should be made to reduce their financial burdens and improve the accessibility of healthcare services.

## 1 Introduction

Health service utilization refers to the actual volume of healthcare services accessed by individuals, serving as a direct measure of the effectiveness and efficiency of delivering comprehensive care for the overall wellbeing of a population (1). The utilization of healthcare services is closely tied to the physical and mental health of migrants, often manifested in their behaviors related to outpatient or inpatient care when faced with discomfort, injuries, or physiological issues (2, 3). As a common metric, health service utilization reflects the societal and economic benefits of healthcare services and indicates the impact of these services on population health outcomes (4, 5). The provision of healthcare services in China is a complex system with multiple levels and channels, with the government taking the lead and medical institutions (including public hospitals, private hospitals and community health service organizations) as the mainstay in providing outpatient and inpatient services. Patients undertake the costs of health service utilization through individual payments, health insurance, and commercial insurance. And the term “underutilization of health services” describes a scenario where individuals or groups fail to make adequate or timely use of available health resources when needed, often resulting in a lack of access to preventive, diagnostic, therapeutic, or rehabilitative services aligned with their health needs.

China, in the past few decades, has experienced a unique challenge related to its internal migrant population due to rapid urbanization. The most commonly accepted definition of this population is from the Census 2000, which describes migrants as individuals residing in a new location for at least 1 year without local household registration. And China’s migrant population reached 376 million, accounting for approximately 26.7% of the total population, and continues to grow (6). Internal migrants often face barriers to accessing healthcare services in their new locations, such as unfamiliarity with local healthcare organizations, service delivery processes, and health insurance policies. Additionally, low health literacy, often due to limited education or language barriers, further complicates their ability to seek timely medical care, exacerbating health risks. As both population migration and the aging process accelerate, there has been a noticeable increase in both the size and proportion of older adults individuals within the migrant population (7, 8). The health challenges of this demographic pose significant pressure on urban management and public health services in regions experiencing an influx of older adults migrants (9). Moreover, addressing the health needs of this growing group is crucial for promoting equitable access to basic public services in urban areas (10). Therefore, it is vital to investigate the healthcare service utilization of migrant older adults to ensure the continued stability and development of urban healthcare services.

A significant portion of China’s internal migrants come from rural areas, and many, including the older adults, migrate to cities for work. These individuals often live-in poor conditions, with low levels of personal and family income, and their financial situation remains bleak. Migrant older adults populations face changes in their economic circumstances, living environments, and social roles (11, 12), and are often considered a vulnerable subgroup within the broader migrant community. They encounter numerous barriers in accessing essential healthcare services (13). Existing studies on healthcare service utilization among migrant older adults primarily rely on national data concerning health insurance, basic public health services, health education, and service satisfaction (14–17), etc. However, much of this research focuses on individual characteristics that are difficult to change, lacks systematic analysis, and often lacks strong theoretical foundations (18–20).

The Anderson model, first proposed by American scholar Ronald M. Andersen in 1968, is a widely used theoretical framework for understanding the factors influencing healthcare service utilization. The model helps researchers grasp the complex mechanisms affecting healthcare access and utilization and provides a basis for designing interventions aimed at improving the accessibility and equity of health services. Anderson’s model incorporates both societal and individual determinants in its analysis. According to the model, health service utilization—whether outpatient or inpatient—is influenced by three key components: predisposing traits, enabling resources, and need factors (PEN) (20). Predisposition traits include factors that exist before an individual seeks healthcare, which can influence a person’s tendency to utilize health services. Enabling resources refer to the conditions that facilitate or hinder access to healthcare, such as income, health insurance coverage, and geographic accessibility to healthcare facilities. Need factors reflect an individual’s health status and their perceived or actual need for healthcare services (21–23). Given its well-established theoretical foundation, Anderson’s model provides a robust framework for analyzing the factors affecting health service utilization, making it particularly useful for examining the healthcare behaviors of migrant older adults.

Xuzhou City, located at the intersection of Jiangsu, Shandong, Anhui, and Henan provinces, has a significant migrant population, including a growing and aging older adults demographic. As of the end of 2022, Xuzhou’s migrant population stood at approximately 1.33 million, with 19.8% aged 60 or older (24). This study aims to assess the current utilization of outpatient and inpatient healthcare services among the migrant older adults population in Xuzhou and to explore the factors influencing this utilization.

## 2 Materials and methods

### 2.1 Study design and participants

A multi-stage random sampling method was employed to select participants from the urban area of Xuzhou between October 2020 and May 2021, focusing on migrant older adults aged 60 years and above. In the first stage, 10 communities were randomly selected from each of the five administrative districts of Xuzhou: Tongshan District, Yunlong District, Gulou District, Quanshan District, and Jiawang District. In the second stage, 12 households were randomly chosen within each community. Finally, in the third stage, one migrant older adults individual was randomly selected from each household to participate in the offline questionnaire survey. A total of 600 questionnaires were distributed, of which 568 were deemed valid after careful scrutiny, yielding an effective response rate of 94.7%.

Inclusion criteria for participants were: migrant older adults aged 60 years and older, who had lived in the local area for at least 1 month, held foreign household registration, and provided informed consent to participate in the survey. Exclusion criteria included individuals in poor health, those with cognitive impairments, and those who were unwilling or unable to complete the questionnaire.

### 2.2 Measurements

According to the definition in social medicine and health management textbooks, outpatient service utilization was assessed by determining whether the respondent had used outpatient services at a health center in the past 2 weeks. Inpatient service utilization was measured by whether the respondent had been hospitalized in the past year. Data were collected through face-to-face questionnaires. Additionally, awareness of and needs for health services were measured across 11 categories, including physical examinations, auxiliary examinations, chronic disease management, health education, interpretation of physical examination results, health guidance, follow-up, and review etc. Both health service awareness and needs were scored on a binary scale (Yes = 1, No = 0), with total scores ranging from 0 to 11.

### 2.3 Variables based on Anderson’s model

The questionnaire was developed following Anderson’s Behavioral Model of Health Service Utilization and through a series of pre-surveys and team discussions. It included three main sections: (1) The first section was predisposition traits, mainly include gender, age, education level, marital status, children quantity, flow range and flow time among participants. (2) The second section was enabling resources, mainly include personal monthly income, pension insurance, access to healthcare services including health insurance availability and time to health centers. (3) The third section was need factors, mainly include self-assessed health status, the number of chronic diseases, sick for the past 2 weeks, their health services awareness, and their health needs.

### 2.4 Statistical analysis

Descriptive statistics were used to calculate the number and percentage of categorical variables, with proportions expressed as percentages for different classifications of each variable.

To explore the associations between various factors and healthcare service utilization, Chi-square tests were employed to assess the factors influencing health service utilization among migrant older adults. Multivariate logistic regressions were selected for analysis, which is particularly useful for identifying the associations between variables (25). Variables that demonstrated statistical significance in the univariate analysis were included in the logistic regression model to explore the determinants of healthcare utilization among the migrant older adults. For non-normally distributed data, such as health service awareness and health service needs, non-parametric tests were used, with results expressed in medians and quartiles. Statistical analyses were conducted using SPSS software (version 22.0), with a significance level of *p* < 0.05 (two-sided) considered statistically significant.

## 3 Results

### 3.1 Participant characteristics

Table 1 shows a balanced distribution in gender, among 568 migrant older adults, with 285 (50.2%) males and 283 (49.8%) females, spanning an age range of 60 and 86 years, with a mean age of (66.9 ± 6.2) years. Within the sample, 434 participants (76.4%) were married. The majority, 366 (64.4%) possessed pension insurance and 496 (87.3%) possessed health insurance.

**Table 1:** Univariate analysis of healthcare utilization.

Features	*N* (%)/*M* (Q_25_, Q_75_)	Outpatient services			Inpatient services		
		*n* (%)	*χ^2^*/Z	*p*	*n* (%)	*χ^2^*/Z	*p*
Predisposition traits							
Gender			0.009	0.926		2.425	0.119
Male	285 (50.2)	37 (13.0)			62 (21.8)		
Female	283 (49.8)	36 (12.7)			47 (16.6)		
Age			3.385	0.184		23.234	**<0.001** ^**a**^
60–69	416 (73.2)	47 (11.3)			61 (14.7)		
70–79	121 (21.3)	21 (17.4)			35 (28.9)		
80–	31 (5.5)	5 (16.1)			13 (41.9)		
Marital status			1.245	0.265		9.506	**0.002**
Have a spouse	434 (76.4)	52 (12.0)			71 (16.4)		
No spouse	134 (23.6)	21 (15.7)			38 (28.4)		
Living alone			1.502	0.22		3.452	0.063
Yes	110 (19.4)	18 (16.4)			28 (25.5)		
No	458 (80.6)	55 (12.0)			81 (17.7)		
Education level			14.808	**0.005**		24.649	**<0.001**
Illiterate	110 (19.4)	19 (17.2)			37 (33.6)		
Elementary school	136 (23.9)	22 (16.2)			28 (20.6)		
Beginning	138 (24.3)	21 (15.2)			25 (18.1)		
High school	84 (14.8)	9 (10.7)			10 (11.9)		
College degree or above	100 (17.6)	2 (2.0)			9 (9.0)		
Children quantity			1.502	0.682		22.216	**<0.001**
0	43 (7.6)	3 (7.0)			3 (7.0)		
1–2	359 (63.2)	47 (13.1)			56 (15.6)		
3–4	136 (23.9)	19 (14.0)			38 (27.9)		
5–	30 (5.3)	4 (13.3)			12 (40.0)		
Flow range			1.462	0.227		0.003	0.96
Ministry	280 (49.3)	36 (12.9)			29 (10.4)		
Out of province	288 (50.7)	59 (20.5)			33 (11.5)		
Flow time (year)			5.911	0.116		1.383	0.71
0–1	145 (25.5)	30 (20.7)			16 (11.0)		
1–	197 (34.7)	42 (21.3)			26 (13.2)		
5–	165 (29.1)	30 (18.2)			22 (13.3)		
10–	61 (10.7)	8 (13.1)			7 (11.5)		
Enable resources							
Personal monthly income (RMB)			5.636	0.228		18.181	**0.001**
0–1,000	133 (23.4)	19 (14.3)			37 (27.8)		
1,000–2,000	165 (29.1)	27 (16.4)			37 (22.4)		
2,001–3,000	120 (21.1)	15 (12.5)			18 (15.0)		
3,001–4,000	59 (10.4)	6 (10.2)			11 (18.6)		
4,000–	91 (16.0)	6 (6.6)			6 (6.6)		
Pension insurance			13.37	**<0.001**		5.74	**0.017**
Yes	366 (64.4)	61 (16.7)			81 (22.1)		
No	202 (35.6)	12 (5.9)			28 (13.9)		
Health insurance			0.233	0.637		6.267	**0.012**
Yes	496 (87.3)	65 (13.1)			103 (20.8)		
No	72 (12.7)	8 (11.1)			6 (8.3)		
Convenience to visit			9.001	**0.003**		9.618	**0.002**
Yes	388 (68.3)	61 (15.7)			88 (22.7)		
No	180 (31.7)	12 (6.7)			21 (11.7)		
Time to health centers (min)			9.631	**0.022**		10.548	**0.014**
0–10	79 (13.9)	15 (19.0)			19 (24.1)		
10–29	191 (33.6)	31 (16.2)			48 (25.1)		
30–59	211 (37.2)	22 (10.4)			30 (14.2)		
60–	87 (15.3)	5 (5.8)			12 (13.8)		
Need factors							
Self-assessed health status			19.743	**<0.001**		46.062	**<0.001**
Bad	176 (31.0)	39 (22.2)			62 (35.2)		
General	179 (31.5)	16 (8.9)			29 (16.2)		
Good	213 (37.5)	18 (8.5)			18 (8.5)		
Number of chronic diseases			38.392	**<0.001**		82.259	**<0.001**
0	211 (37.2)	8 (3.8)			10 (4.7)		
1	198 (34.9)	31 (15.7)			42 (21.2)		
2	110 (19.3)	17 (15.5)			28 (25.5)		
3–	49 (8.6)	17 (34.7)			29 (59.2)		
Sick for the past 2 weeks			131.502	**<0.001**		42.857	**<0.001**
Yes	105 (18.5)	49 (46.7)			44 (41.9)		
No	463 (81.5)	24 (5.2)			65 (14.0)		
Health services awareness	2.5 (0, 7)	1 (0, 6)	2.112	**0.035**	2 (0, 6)	1.227	0.22
Health service needs	2.5 (0, 10)	6 (0, 11)	−2.719	**0.007**	5 (1, 11)	−2.779	**0.006**

a The bold data indicates that *p* value less than 0.05.

### 3.2 Univariate analysis of healthcare services utilization

Table 1 also illustrates the healthcare service utilization among the 568 migrant older adults participants. A total of 73 participants (12.9%) reported using outpatient services at health centers in the past 2 weeks. The analysis revealed significant differences in outpatient service utilization based on several variables. In the predisposing traits category, only education level showed statistical significance. In the enabling resources category, factors such as pension insurance, convenience to visit, and time to health centers were significant. Finally, in the need factors category, self-assessed health status, the number of chronic diseases, sick for the past 2 weeks, and health service needs were all significantly associated with outpatient service utilization (*p* < 0.05).

Regarding inpatient services, 109 participants (19.2%) reported being hospitalized in the past year. The analysis showed significant differences in inpatient service utilization based on age, marital status, education level, and number of children in the predisposing traits category. In the enabling resources category, variables such as personal monthly income, pension insurance, health insurance, convenience to visit, and time to health centers were significantly associated with inpatient service utilization. The need factors of self-assessed health status, the number of chronic diseases, sick for the past 2 weeks, and health service needs also showed statistical significance (*p* < 0.05).

### 3.3 Multivariate logistic regression analysis

#### 3.3.1 Factors influencing the outpatient services utilization

Table 2 demonstrates a notably diminished outpatient services utilization among highly educated migrant older adults (OR = 0.13, 95% CI: 0.03, 0.71) in compared to their counterparts with limited education levels in predisposing traits. Among the enabling resources, the likelihood of utilizing outpatient services was significantly higher for those with pension insurance (OR = 4.34, 95% CI: 1.86, 10.11) and those reporting high convenience to visit (OR = 2.85, 95% CI: 1.31, 6.22). Conversely, participants whose travel time to health centers was 60 min or more were significantly less likely to utilize outpatient services (OR = 0.19, 95% CI: 0.15, 0.80) compared to those with a travel time of less than 10 min. In terms of need factors, participants in good self-assessed health were significantly less likely to utilize outpatient services (OR = 0.44, 95% CI: 0.24, 0.82) compared to those with poorer self-assessed health. Conversely, those who had been sick in the past 2 weeks (OR = 14.91, 95% CI: 7.32, 30.39) and those with higher health service needs (OR = 1.08, 95% CI: 1.02, 1.14) were significantly more likely to utilize outpatient services.

**Table 2:** The logistic regression of outpatient service utilization.

Features	*p*	OR (95% CI)
Predisposition traits		
Education level (Ref. Illiteracy)		
Elementary school	0.920	1.05 (0.44, 2.46)
Beginning	0.249	1.68 (0.70, 4.05)
High school	0.700	1.24 (0.42, 3.70)
College degree or above	**0.018** ^**a**^	0.13 (0.03, 0.71)
Enable resources		
Pension insurance (Ref. No)	**0.001**	4.34 (1.86, 10.11)
Convenience to visit (Ref. No)	**0.008**	2.85 (1.31, 6.22)
Time to health centers (min) (Ref. <10)		
10–29	0.606	0.79 (0.32, 1.96)
30–59	0.174	0.52 (0.20, 1.34)
60–	**0.023**	0.19 (0.05, 0.80)
Need factors		
Self-assessed health status (Ref. Bad)		
General	**0.003**	0.39 (0.21, 0.73)
Good	**0.010**	0.44 (0.24, 0.82)
Number of chronic diseases (Ref. 0)		
1	0.103	2.26 (0.85, 6.02)
2	0.773	1.18 (0.39, 3.62)
3–	0.082	3.08 (0.87, 10.95)
Sick for the past 2 weeks (Ref. No)	**<0.001**	14.91 (7.32, 30.39)
Health services awareness	0.708	0.98 (0.89, 1.08)
Health service needs	**0.007**	1.08 (1.02, 1.14)

a The bold data indicates that *p* value less than 0.05.

#### 3.3.2 Factors influencing the inpatient services utilization

Table 3 reveals that migrant older adults aged 80 and above demonstrated significantly elevated inpatient health services utilization (OR = 3.38, 95% CI: 1.23, 9.29) compared to their counterparts aged 60–69 years in predisposing traits. Among the enabling resources, pension insurance (OR = 2.06, 95% CI: 1.09, 3.92), health insurance coverage (OR = 2.88, 95% CI: 1.22, 6.84), and convenient access to visit (OR = 2.70, 95% CI: 1.45, 5.03) were significantly associated with higher inpatient service utilization. Conversely, those with travel times 60 min or more (OR = 0.37, 95% CI: 0.13–0.96) were significantly less likely to utilize inpatient services compared to those with shorter travel times. Regarding the need factors, participants in good self-assessed health (OR = 0.17, 95% CI: 0.10, 0.30) were significantly less likely to utilize inpatient services compared to those in poor self-assessed health. Conversely, those with three or more chronic diseases (OR = 29.15, 95% CI: 12.42, 68.40), those who had been sick in the past 2 weeks (OR = 2.08, 95% CI: 1.13, 3.80), and those with higher health service needs (OR = 1.07, 95% CI: 1.00, 1.13) were significantly more likely to utilize inpatient services.

**Table 3:** The logistic regression of inpatient service utilization.

Features	*p*	OR (95% CI)
Predisposition traits		
Age (Ref. 60–69)		
70–79	0.430	1.29 (0.69, 2.24)
80–	**0.018** ^**a**^	3.38 (1.23, 9.29)
Marital status (Ref. No spouse)	0.870	0.95 (0.50, 1.81)
Education level (Ref. Illiterate)		
Elementary school	**0.030**	0.45 (0.22, 0.93)
Beginning	0.085	0.49 (0.22, 1.10)
High school	0.164	0.47 (0.16, 1.36)
College degree or above	0.586	0.73 (0.23, 2.31)
Children quantity (Ref. 0)		
1–2	0.789	1.25 (0.25, 6.26)
3–4	0.644	1.48 (0.28, 7.81)
5–	0.253	3.00 (0.46, 19.65)
Enable resources		
Personal monthly income (RMB) (ref. 0–1,000)		
1,000–2,000	0.322	1.44 (0.70, 2.96)
2,001–3,000	0.922	0.96 (0.40, 2.29)
3,001–4,000	0.267	1.87 (0.62, 5.60)
4,000–	0.114	0.37 (0.11, 1.27)
Pension insurance (Ref. No)	**0.027**	2.06 (1.09, 3.92)
Health insurance (Ref. No)	**0.016**	2.88 (1.22, 6.84)
Convenience to visit (Ref. No)	**0.002**	2.70 (1.45, 5.03)
Time to health centers (min) (Ref. <10)		
10–29	0.410	0.72 (0.33, 1.57)
30–59	**0.038**	0.42 (0.18, 0.95)
60–	**0.044**	0.37 (0.13, 0.96)
Need factors		
Self-assessed health status (Ref. Bad)		
General	**<0.001**	0.36 (0.22, 0.59)
Good	**<0.001**	0.17 (0.10, 0.30)
Number of chronic diseases (Ref. 0)		
1	**<0.001**	5.41 (2.63, 11.13)
2	**<0.001**	6.86 (3.19, 14.77)
3–	**<0.001**	29.15 (12.42, 68.40)
Sick for the past 2 weeks (Ref. No)	**0.018**	2.08 (1.13, 3.80)
Health service needs	**0.042**	1.07 (1.00, 1.13)

a The bold data indicates that *p* value less than 0.05.

## 4 Discussion

### 4.1 The rate of healthcare services utilization among the migrant older adults in Xuzhou is comparatively low and requires improvement

The study found that, among the 568 participants, only 73 individuals (12.9%) utilized outpatient services within the past 2 weeks. This rate is considerably lower than the average outpatient service utilization for migrant populations in the province (20.42%) (26, 27), and also below the national average for older adults populations (20.9%) (28, 29). In the past year, inpatient services were utilized by 109 migrant older adults (19.2%), which is again lower than the rates reported in most recent studies (29.9%) (30, 31). This discrepancy can be attributed to the fact that a large proportion of the sample (73.2%) consisted of migrant older adults aged 60–69, who generally enjoy relatively good health and exhibit fewer evident healthcare needs, thereby lowering their utilization rates (32).

Additionally, limited health awareness among the migrants and a lack of understanding of healthcare services at their destination may lead them to rely on purchasing medication from pharmacies or even avoid seeking medical attention when experiencing discomfort (33). These factors contribute to the relatively low healthcare service utilization observed in this study. The findings suggest, on one hand, a lack of disease knowledge and insufficient attention to personal health among the migrant older adults in Xuzhou, pointing to the need for improved health education. On the other hand, they highlight the limited understanding of available health services, underscoring the importance of strengthening health promotion efforts targeting migrant populations. Furthermore, it is recommended that migrant older adults be included in health management through community health service centers in order to promote more effective health services utilization.

### 4.2 Multiple factors influence the utilization of outpatient health services among migrant older adults

Among the three characteristics in Anderson’s model, enabling resources and need factors exert the most significant influence on outpatient service utilization among migrant older adults. Predisposing traits play a limited role, with one exception: migrant older adults with higher education levels (college or above) tend to develop a stronger sense of health awareness (34, 35), which correlates with a reduced likelihood of utilizing outpatient services. These individuals often possess basic medical knowledge and have a better understanding of their health conditions (16), leading them to prefer purchasing medications from pharmacies or using medicines brought from their hometowns to manage minor health issues (36).

At the enabling resources level, outpatient service utilization is higher among those with pension insurance and convenient access to healthcare services. Conversely, migrant older adults who need to travel more than 60 min to reach a health center show significantly lower utilization rates. Pension insurance plays a pivotal role in providing financial security, supporting basic healthcare needs (16), and promoting the utilization of outpatient services. However, the underutilization of outpatient services among those with long travel times highlights the critical impact of accessibility and convenience on healthcare utilization (21–23). The mobility of migrant older adults across provinces or regions introduces challenges such as varying medical policies, lifestyle differences, language barriers, and limited access to resources, prompting many to rely on self-medication or informal care for minor ailments (37, 38). Implementing comprehensive health management for migrant older adults could help ensure more equitable and efficient access to healthcare services.

Previous studies have also emphasized the predictive power of need factors in determining healthcare service utilization, particularly among the older adults (39–41). For outpatient services, good self-assessed health status is associated with lower utilization, while recent illness (within the past 2 weeks) increases the demand for healthcare services. Migrant older adults in good self-assessed health are less likely to engage with healthcare services, as their health status accurately reflects their overall wellbeing (42–44). Migrant older adults with poor self-assessed health status tend to have a subjective perception of their own health status (45), thereby increasing the likelihood of accepting outpatient medical services. Migrant older adults with high health service needs typically exhibit varying degrees of physical or mental impairment or are in a suboptimal state of health, leading to an increased inclination to utilize healthcare services (46). Moreover, the attribute of mobility can further exacerbate concerns about their health status among migrant older adults, potentially resulting in a psychological burden and consequently driving them toward seeking healthcare services (47). Consequently, the impact of the mental health and health awareness of migrant older adults people in the field of health can be targeted in subsequent research.

### 4.3 Multiple factors influence the utilization of inpatient health services among migrant older adults

In addition to outpatient services, predisposing traits significantly influence inpatient service utilization, particularly among migrant older adults aged 80 and above, who demonstrate much higher utilization rates than their younger counterparts (aged 60–69). This increase is likely due to the natural aging process and the associated decline in physiological functioning (48, 49) as well as the inverse relationship between age and overall health status (49, 50), leading to a greater demand for inpatient care (50, 51). With advancing age, the prevalence of chronic diseases rises (52, 53), further increasing the need for inpatient services. Previous research has documented the high prevalence of chronic diseases among the older adults in Xuzhou (54), and migrant older adults with multiple chronic conditions exhibit significantly higher utilization of inpatient services compared to those without such ailments (55). Chronic diseases such as hypertension and diabetes, which often lead to debilitating complications, profoundly affect the quality of life for migrant older adults and drive their reliance on inpatient care (56, 57).

Among enabling resources, limitations in Medicare coverage and pension income restrict the utilization of residential health services by migrant older adults. This study highlights the significant impact of health insurance coverage on healthcare utilization (58). The low reimbursement rates for hospitalization expenses at the destination, coupled with complex reimbursement procedures and limitations on eligible services, create barriers to inpatient healthcare access for migrant older adults (49, 59). Socioeconomic factors also contribute to disparities in healthcare utilization, as migrant older adults often face financial instability due to inconsistent incomes (60, 61). However, pension insurance can reduce reliance on offspring for healthcare costs, especially for hospitalization, which can incur substantial expenses (26). Conversely, migrant older adults without pension or health insurance are less likely to utilize healthcare services. To address these challenges, enhancing the development of medical insurance information systems is crucial for facilitating cross-regional medical expense settlements. Additionally, efforts should be made to promote commercial health insurance as a supplementary option for migrant older adults. Expanding the scope of reimbursement and increasing hospitalization coverage rates can alleviate financial burdens and ultimately improve healthcare utilization among this population (62).

### 4.4 Limitation

This study is subject to several limitations that should be acknowledged. First, the cross-sectional design limits the ability to establish causal relationships regarding healthcare service utilization among the migrant older adults. Second, this study’s geographic focus on Xuzhou City may introduce regional biases, as the findings are influenced by the specific characteristics and healthcare landscape of this area. Third, we did not collect data on key variables such as social integration, familiarity with the healthcare system in the destination, or the existence of personal health records, and their absence limits the depth of our analysis regarding the influences on healthcare services utilization. Despite these limitations, our study provides a foundation for future research and offers valuable insights for improving the utilization of healthcare services.

## 5 Conclusion

Overall, compared with the migrant population and the older adults in the non-migrant population, healthcare service utilization among the migrant older adults in Xuzhou is suboptimal. The study identified several key factors influencing healthcare service utilization, including age, education level, possession of pension and health insurance, ease of access to healthcare facilities, travel time to health centers, health status, recent illness (within the past 2 weeks), number of chronic diseases, and perceived need for healthcare services. To improve healthcare access for migrant older adults, it is essential to consider a multifaceted approach. This should include policy support and assistance, reducing the medical and economic burdens faced by this population, improving the accessibility and convenience of healthcare organizations, and prioritizing health education and promotion efforts. By addressing these factors, policymakers and healthcare providers can work toward enhancing the healthcare experiences and outcomes for migrant older adults populations.

## Data Availability

The raw data supporting the conclusions of this article will be made available by the authors without undue reservation.
